# Differential control of CXCR4 and CD4 downregulation by HIV-1 Gag

**DOI:** 10.1186/1743-422X-5-23

**Published:** 2008-02-11

**Authors:** Rajeshwari R Valiathan, Marilyn D Resh

**Affiliations:** 1 Cell Biology Program, Memorial Sloan-Kettering Cancer Center, New York, USA and Graduate Program in Biochemistry, Cell and Molecular Biology, Weill Graduate School of Medical Sciences of Cornell University, New York, USA; 2 University of Michigan, Life Sciences Institute, Ann Arbor, USA

## Abstract

**Background:**

The ESCRT (endosomal sorting complex required for transport) machinery functions to sort cellular receptors into the lumen of the multivesicular body (MVB) prior to lysosomal degradation. ESCRT components can also be recruited by enveloped viruses to sites of viral assembly where they have been proposed to mediate viral egress. For example, HIV-1 budding is dependent on Gag-mediated recruitment of the cellular ESCRTs-I, -III, AIP1/Alix and Vps4 proteins. Viral recruitment of ESCRT proteins could therefore impact on host cell processes such as receptor downregulation.

**Results:**

Here we show that downregulation of the HIV-1 co-receptor, CXCR4, by its ligand SDF-1, is ESCRT-I dependent. Expression of HIV-1 Gag attenuated downregulation of CXCR4, resulting in accumulation of undegraded receptors within intracellular compartments. The effect of Gag was dependent on an ESCRT-I interacting motif within the C-terminal p6 region of Gag. In contrast, PMA-induced downregulation of the HIV-1 receptor CD4 was independent of ESCRT-I and Vps4; HIV-1 Gag had no effect on this process.

**Conclusion:**

These results establish that the HIV-1 receptor, CD4, and co-receptor, CXCR4 are differentially regulated by ESCRT proteins. HIV-1 Gag selectively modulates protein sorting at the MVB, interfering with ESCRT-I dependent but not ESCRT-I independent processes.

## Background

Many transmembrane receptors that are destined for lysosomal degradation are directed through the multivesicular body (MVB) sorting pathway [1,2]. Internalized receptors are sorted into endosomal membrane invaginations, which then pinch off to form intralumenal vesicles within the MVB. This process is initiated by the ubiquitin-binding protein, Hrs, which recruits the endosomal sorting complex required for transport (ESCRT)-I to endosomal membranes by directly interacting with the ESCRT-I component, TSG101 (Tumor Susceptibility Gene 101) [3]. Following ESCRT-I recruitment, ESCRTs-II and III are sequentially localized to the endosomal membrane [1]. These complexes bind ubiquitylated receptors and are required for receptor sorting into the lumen of the MVB. The AAA type ATPase Vps4 then facilitates the disassembly of the ESCRT complexes prior to membrane fission, thereby ensuring that these complexes are available for further rounds of protein sorting [4].

In many respects, vesicle formation in the MVB is topologically identical to viral budding at the plasma membrane: both processes involve budding away from the cytosol. Most enveloped viruses have evolved strategies to gain access to cellular ESCRTs in order to mediate virion egress from the infected cell [5]. For example, HIV-1 recruits ESCRT complexes to sites of viral assembly through direct interactions between the Gag polyprotein and two cellular ESCRT proteins: TSG101 and AIP1/Alix [6-9]. Depletion of TSG101 or introduction of dominant negative mutants of AIP1/Alix arrests HIV-1 budding at a late stage and blocks viral particle release [7,10]. Likewise, depletion of TSG101 and other ESCRT components inhibits lysosomal downregulation of ligand-activated growth factor receptors, such as the EGF Receptor (EGFR) [11-13]. Given the fact that HIV-1 budding and EGFR downregulation both require ESCRT function, it is logical to question whether there is competition for cellular ESCRT components when both processes occur in the same cell at the same time.

We have previously shown that expression of HIV-1 Gag decreases the rate of EGF induced EGFR degradation [14]. This effect is dependent on the presence of an intact TSG101-binding sequence within the Gag polyprotein. As a result, activated EGFR accumulates in late endosomal compartments and Gag expressing cells exhibit higher levels of activated MAP Kinase. These findings indicate that HIV-1 Gag impinges upon the normal function of cellular ESCRT complexes during EGFR downregulation. In order to determine whether downregulation of other receptors is sensitive to HIV-1 Gag expression, we have now investigated the kinetics of lysosomal downregulation of CD4 and CXCR4, in the presence and absence of Gag. CD4 and CXCR4 function as the receptor and co-receptor respectively for the entry of HIV-1 X4 variants into target cells [15]. Regulation of the cell surface levels of these two proteins is critically important for HIV-1 pathogenesis.

CXCR4 is a seven transmembrane G-Protein Coupled Receptor (GPCR) expressed on the cell surface of various leukocytes such as neutrophils, monocytes and lymphocytes [16,17]. The ligand for CXCR4 is the chemokine stromal cell-derived factor (SDF-1), which regulates the movement of leukocytes during their development, homeostasis and inflammatory response [18]. Upon SDF-1 binding, CXCR4 is rapidly phosphorylated by GPCR kinase (GRK) and internalized via clathrin-coated pits [19]. SDF-1-bound CXCR4 is also monoubiquitylated by the Nedd4 like E3 Ub ligase AIP4 [20]; this promotes sorting of CXCR4 into the internal vesicles of the MVB prior to lysosomal degradation. While Hrs and Vps4 have been implicated in the lysosomal degradation of monoubiquitylated CXCR4 [20], no study has determined whether the ESCRT complexes play a role in this process. Hrs and Vps4 have been shown to function in ESCRT-dependent [3] as well as ESCRT-independent [21] pathways of receptor sorting. Identifying which of the two Hrs-dependent pathways is functional in the lysosomal downregulation of CXCR4 is important since this may also have implications for the trafficking of this receptor in HIV-1 infected cells.

CD4 is a transmembrane glycoprotein that is expressed on the surface of a subset of T cells as well as monocytes and macrophages. It normally functions as a signal transducer during T cell activation. Antigens, mitogens and PKC modulators such as PMA induce internalization of CD4, thereby regulating its cell surface expression [22,23]. PMA induces phosphorylation of CD4, resulting in its rapid internalization from the cell surface and degradation in lysosomes [24,25]. Similarly, the HIV-1 Nef protein induces endocytosis and lysosomal degradation of CD4 [26]. Both PMA and Nef-induced CD4 internalization are dependent on the clathrin adaptor protein AP-2 [27]. The sorting of CD4 from early endosomes into late endosomes/MVBs during Nef-induced downregulation has been shown to be dependent on the interaction of Nef with the β subunit of the coatomer protein-(COP)-1 complex and ARF1 [28,29]. How this sorting step occurs during PMA-induced CD4 downregulation and whether ESCRT complexes are involved is not known.

Given the fundamental importance of both CXCR4 and CD4 for both normal cell physiology and HIV-1 biology, we have examined the role of ESCRT I in downregulation of these two cellular proteins. SDF-1-induced downregulation of CXCR4 and PMA-induced downregulation of CD4 were monitored in cells depleted of endogenous TSG101 using siRNA directed against TSG101. We also monitored CXCR4 and CD4 downregulation in cells expressing HIV-1 Gag. Our findings indicate that SDF-1-induced CXCR4 downregulation is defective when ESCRT-I function is impaired, while PMA-induced CD4 downregulation is not. Consequently, the expression of HIV-1 Gag attenuated CXCR4 downregulation, while having little to no effect on CD4 downregulation. These results have important implications for the endocytic trafficking of CD4 and CXCR4 in normal as well as HIV-1 infected cells.

## Results

### SDF-1 induced CXCR4 downregulation is ESCRT-I dependent

**Figure 1:**
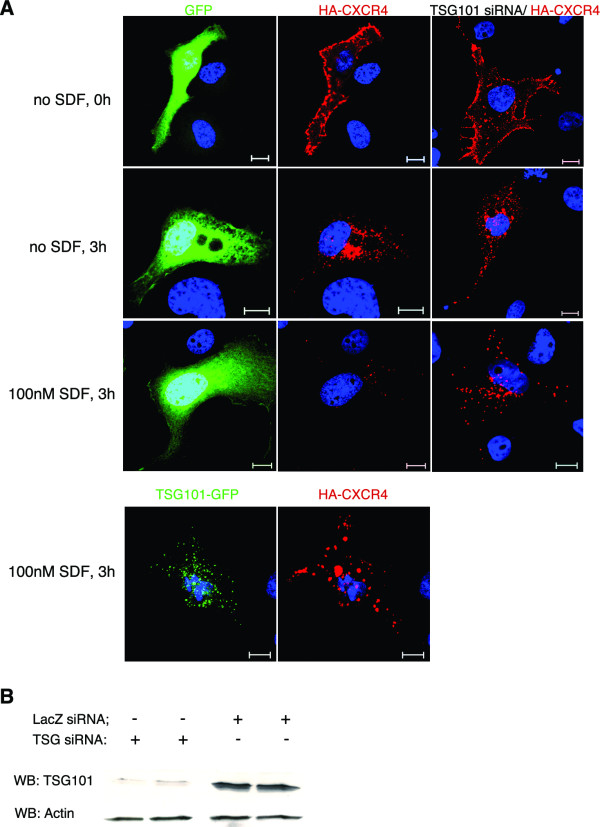
**SDF-1 induced CXCR4 downregulation is TSG101-dependent**. (A) COS-1 cells co-transfected with HA-tagged CXCR4 and either GFP, TSG101-GFP, or siRNA directed against TSG101, as indicated, were incubated with an anti-HA antibody for 1 hour on ice. Cells were then either fixed and stained with a secondary antibody to detect cell surface CXCR4 (first row), or incubated in DMEM/10% FBS without SDF (second row) or with 100 nM SDF (third and fourth rows) for 3 hours at 37°C prior to fixation, permeabilization and secondary-antibody staining. The cells were then analysed for GFP fluorescence (green), or CXCR4 expression (red). Blue represents nuclear staining. Scale bars = 10 μm. (B) Immunoblot of COS-1 cells co-transfected with HA-tagged CXCR4 and siRNA directed against either TSG101 or LacZ. Top panel, TSG101 Western blot; bottom panel, actin Western blot.

Previous studies have established that SDF-1 induces internalization, endosomal trafficking and lysosomal degradation of CXCR4 and HA-tagged CXCR4 in a variety of cell types [19,20,30]. To study the role of TSG101 in CXCR4 downregulation, we used transfected COS-1 cells co-expressing GFP and HA-tagged CXCR4. HA-CXCR4 has previously been shown to be a valid marker for CXCR4 trafficking and degradation in COS-1 cells [19,20]. The plasma membrane population of HA-CXCR4 was first labeled using an anti-HA antibody. Cells were then incubated with or without SDF-1 for 3 hours. In the absence of SDF-1, a considerable amount of internalization of HA-CXCR4 was observed (Fig 1A). This observation confirms previous reports and likely reflects a combination of constitutive endocytosis [31] and antibody-induced, ligand-independent endocytosis of CXCR4 [17]. HA-CXCR4 that was internalized in the absence of SDF-1 appeared in punctate, endosomal structures and remained undegraded. In contrast, cells that were incubated in the presence of SDF-1 clearly exhibited a loss in receptor signal, confirming that SDF-1 induces degradation of HA-CXCR4.

In order to determine whether SDF-1 induced HA-CXCR4 downregulation is dependent on the ESCRT-I complex, cells were depleted of the critical ESCRT-I component, TSG101. Addition of siRNA directed against TSG101 resulted in 80% knockdown of endogenous TSG101 levels (Fig. 1B). SDF-1 induced HA-CXCR4 degradation was significantly attenuated in TSG101-deficient cells (Fig. 1A), as indicated by the retention of receptors in punctate structures even after 3 hours of incubation with SDF-1. An alternative method to interfere with TSG101 function was also implemented. Overexpression of full length TSG101 has been shown to inhibit ESCRT-I function and block EGF-induced EGFR downregulation [14,32]. COS-1 cells overexpressing TSG101 also exhibited attenuated HA-CXCR4 degradation (Fig. 1A). These data indicate that HA-CXCR4, like EGFR, is dependent on TSG101 function for SDF-1 mediated degradation.

### Expression of HIV-1 Gag inhibits HA-CXCR4 degradation in a late-domain dependent manner

Recruitment of ESCRT-I complexes to sites of viral assembly by HIV-1 Gag mediates the separation of viral and host membranes during the virus release process. We have previously shown that Gag expression results in the functional depletion of ESCRT-I complexes. EGF induced EGFR downregulation, an ESCRT-I dependent process, was attenuated in HIV-1 Gag expressing cells [14]. Since SDF-1 induced degradation of HA-CXCR4 also appears to be ESCRT-I dependent, we hypothesized that HA-CXCR4 degradation would also be attenuated in HIV-1 Gag expressing cells.

**Figure 2:**
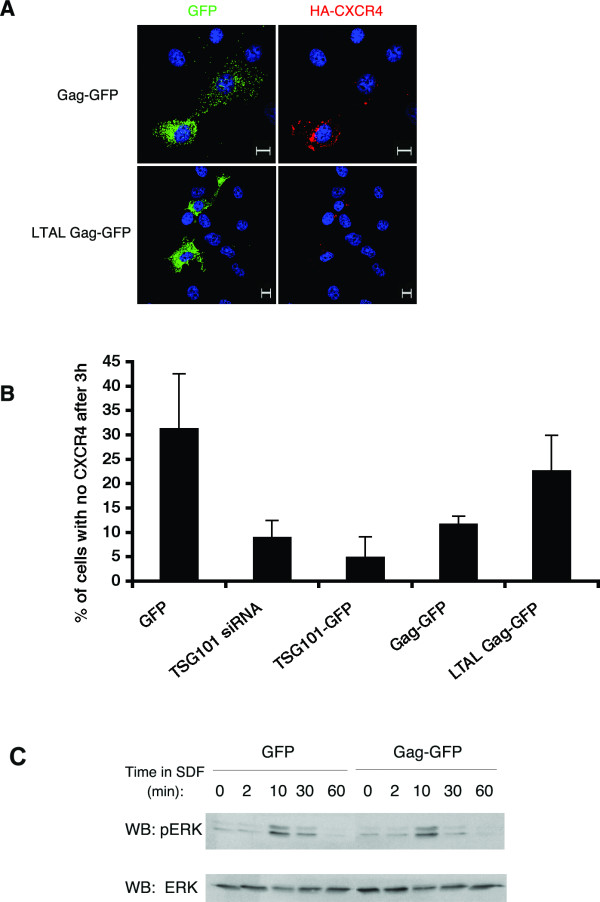
**HIV-1 Gag attenuates CXCR4 degradation in COS-1 cells**. (A) COS-1 cells co-expressing HA-tagged CXCR4 (red) and either wild type (top panels) or LTAL, a PTAP mutant form (bottom panels) of Gag-GFP (green) were treated as described in Figure 1A. Only cells that were incubated for 3 hours in the presence of SDF are shown. Scale bars = 10 μm. (B) The percent of COS-1 cells that had no visible HA-CXCR4 remaining after 3 hours of incubation with SDF was determined for each of the conditions described in Figs. 1 and 2A. Data shown represents the mean ± SD of 2 or more independent experiments. 50 to 100 cells were analyzed per experiment. (C) COS-1 cells co-expressing HA-CXCR4 and GFP or Gag-GFP were lysed at various times after SDF treatment and analyzed by SDS-PAGE and Western blotting with anti-pERK1/2 and anti-ERK antibodies, as indicated. One representative immunoblot (out of three independent experiments) is shown.

To test this hypothesis, COS-1 cells were co-transfected with plasmids encoding HA-CXCR4 and Gag-GFP (GFP-tagged HIV-1 Gag). Cells expressing wild type HIV-1 Gag-GFP exhibited attenuated HA-CXCR4 degradation (Fig 2A). This effect of Gag was dependent on its TSG101-interacting PTAP sequence, located within the C-terminal p6 region of the Gag polyprotein. Cells expressing a Gag PTAP mutant efficiently degraded HA-CXCR4 (Fig. 2A).

HA-CXCR4 degradation efficiencies were quantitated in cells expressing various GFP-tagged constructs. HA-CXCR4 degradation was decreased 3–6 fold in cells expressing TSG101-GFP or Gag-GFP, compared to cells expressing GFP (Fig 2B). A similar effect was noted in cells depleted of TSG101. In contrast, CXCR4 degradation in cells expressing the late domain mutant, LTAL Gag-GFP was nearly equivalent to that of control cells (Fig 2B). These results suggest that expression of wild type HIV-1 Gag interferes with the function of endogenous TSG101 and/or ESCRT-I machinery, resulting in increased accumulation of internalized, undegraded HA-CXCR4, following SDF-1 treatment.

We next examined whether accumulation of intracellular HA-CXCR4 caused alterations in SDF-1 mediated signaling. GPCRs are known to be rapidly desensitized after ligand binding and internalization. One would therefore predict that accumulation of intracellular, inactivated receptors would not alter signaling. To test this hypothesis, the time course of pERK formation, a downstream readout of SDF-1 mediated CXCR4 signaling, was monitored. As depicted in Figure 2C, cells expressing Gag-GFP exhibited identical kinetics and levels of pERK production when compared to cells expressing GFP. Thus, accumulation of intracellular HA-CXCR4 did not result in altered SDF-1 induced CXCR4 signaling in Gag expressing cells.

### HIV-1 Gag attenuates SDF-1 induced downregulation of endogenous CXCR4 in Jurkat T cells

In transfected COS-1 cells, both HA-CXCR4 and HIV-1 Gag were exogenously expressed at high levels. We have previously shown that the levels of Gag expressed under a CMV promoter (as in the experiments above) are comparable to HIV-1 LTR-driven Gag expression levels in COS-1 cells and might therefore be representative of the levels of Gag in an HIV-1 infected cell [14]. In order to examine the effects of Gag expression on endogenous CXCR4, we monitored the kinetics of SDF-1 induced downregulation of CXCR4 in Jurkat T cells. Jurkat cells express endogenous CXCR4, and are authentic targets of HIV-1 infection *in vivo*. Therefore, studying the effects of HIV-1 Gag expression on CXCR4 downregulation kinetics in these cells should provide insight into the physiologic processes occurring during HIV-1 infection.

**Figure 3:**
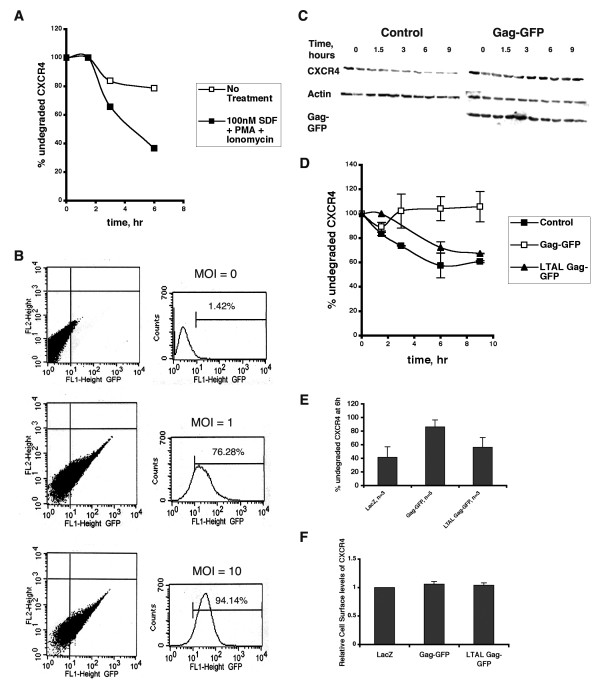
**HIV-1 Gag attenuates SDF-induced CXCR4 downregulation in Jurkat T cells**. (A) Jurkat T cells were pre-treated with cycloheximide, then incubated in the presence (filled squares) or absence (open squares) of SDF, PMA and ionomycin for the indicated times. At each time point, cells were lysed and analysed by SDS-PAGE and Western blotting with an anti-CXCR4 polyclonal antibody. Western blots were quantitated and amount of CXCR4 remaining at each time point was determined as a percent of amount of CXCR4 at 0 hour. Data from one representative experiment (out of three) is shown. (B) Jurkat T cells were transduced with Gag-GFP encoding lentiviruses at the indicated MOIs. 48 hours post transduction, cells were analyzed for GFP fluorescence by flow cytometry. The % of GFP positive cells is indicated for each MOI. (C) A representative gel depicting CXCR4 levels in Jurkat T cells transduced with lentiviruses encoding wild-type Gag-GFP and treated as described in (A) is shown. Control represents untransduced cells. (D) Quantitation of the gel shown in (C). Additionally, degradation of CXCR4 in Jurkat T cells transduced with lentiviruses encoding LTAL Gag-GFP is shown. Error bars represent standard deviation between duplicates at each time point. (E) Jurkat T cells were transduced with lentivirus encoding LacZ (control), Gag-GFP or LTAL Gag-GFP. Data shown represents the mean ± SD of % undegraded CXCR4 remaining after 6 hours of incubation with SDF, PMA and ionomycin. (F) Cell surface levels of CXCR4 in Jurkat T cells expressing LacZ, Gag-GFP or LTAL Gag-GFP were determined 48 hours post transduction by flow cytometric analysis of cells stained with a biotinylated anti-CXCR4 antibody and Streptavidin-PE. Data shown represents the mean ± SD of surface CXCR4 levels relative to the control, from two independent experiments.

Previous studies have shown that T cells have large intracellular stores of CXCR4 that can be mobilized by treating the cells with PMA and ionomycin [17]. Indeed, in order to observe SDF-1 induced CXCR4 degradation in Jurkat cells, we needed to inhibit the synthesis of new receptors continuously with cycloheximide and incubate the cells with SDF-1, PMA and ionomycin (Fig. 3A). Efficient expression of HIV-1 Gag was achieved by transducing Jurkat cells with Gag-GFP-encoding lentiviruses. At a multiplicity of infection (MOI) of 10, over 90% of the cells expressed Gag-GFP (Fig. 3B). Incubation of Gag-GFP expressing Jurkat cells with SDF-1, PMA and ionomycin revealed that downregulation of endogenous CXCR4 was clearly attenuated by expression of wt Gag-GFP (Fig. 3C,D,E). In contrast, cells expressing the late domain mutant, LTAL Gag-GFP, exhibited CXCR4 degradation rates more similar to the LacZ control (Fig 3D,E). Notably, cell surface levels of CXCR4 at steady state were not altered in HIV-1 Gag expressing cells (Fig. 3F). Thus, experiments in Jurkat cells reveal that expression of HIV-1 Gag attenuates downregulation of endogenous CXCR4 in the presence of SDF-1, PMA and ionomycin. These data are similar to those reported in Figure 2, for SDF-I induced HA-CXCR4 degradation.

### PMA-induced lysosomal degradation of CD4 is independent of TSG101 and Vps4

CD4 is a cell surface transmembrane glycoprotein whose endocytic trafficking is of great significance to the HIV-1 life cycle. Previous studies have shown that PMA induces internalization and lysosomal degradation of CD4. However, the role of the ESCRT complexes in CD4 downregulation is not known, nor is it known how, or if HIV-1 Gag expression affects this process.

Previous studies have quantitated CD4 degradation kinetics by monitoring levels of metabolically (pulse) labeled CD4 over time in untreated and PMA-treated cells [24,33,34]. Pulse labeled CD4 has been shown to proceed to the cell surface via the secretory pathway within 30–60 minutes after synthesis, internalize via endocytosis, and undergo degradation in lysosomes [34]. We were unable to immunoprecipitate endogenous CD4 from Jurkat T cells using a wide range of available anti-CD4 antibodies. We therefore examined PMA-induced downregulation of exogenously expressed CD4, which was readily radiolabeled and immunoprecipitated using a monoclonal anti-CD4 (Leu3a) antibody. Trafficking of exogenous CD4 has been shown to accurately represent that of endogenous CD4 [35], and we have previously shown that CD4 is trafficked to the cell surface in transfected COS-1 cells [36,37].

**Figure 4:**
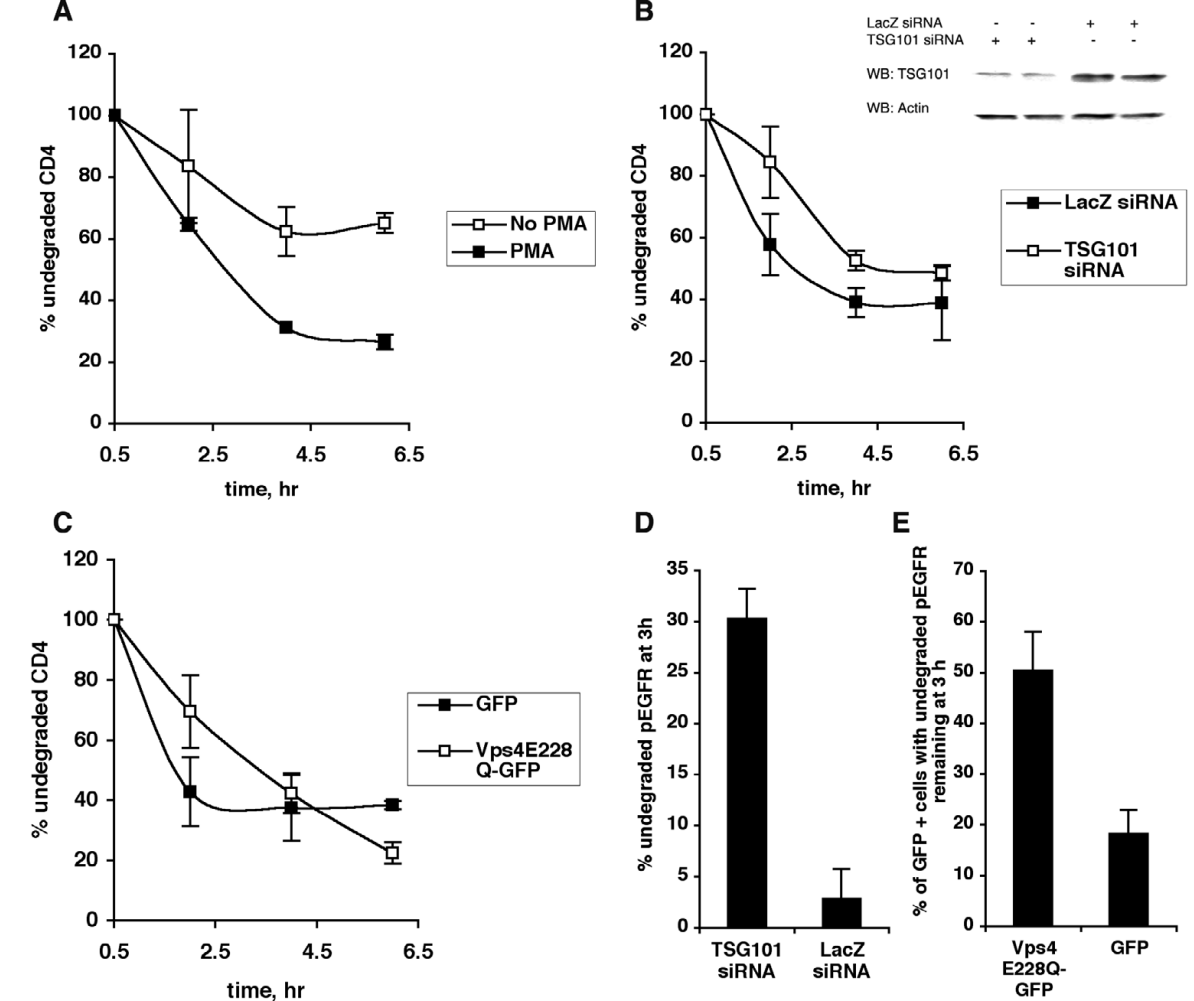
**PMA-induced CD4 downregulation is TSG101 and Vps4 independent**. (A) COS-1 cells transfected with CD4 were labeled with trans-^35^S-label for 10 minutes, then chased in non-radioactive medium in the presence or absence of 50 ng/ml PMA for the indicated times. At each time point, cells were lysed and radioactive CD4 was analyzed by immunoprecipitation, SDS-PAGE and phosphorimaging. The amount of CD4 remaining at each time point was determined as a percent of the amount of CD4 at the first chase time point, ie 0.5 hours. Data from one representative experiment (out of three) is shown. Error bars represent standard deviation between duplicates for each condition at each time point. (B) COS-1 cells co-transfected with CD4 and siRNA against TSG101 or LacZ were treated as described in (A). Data from one representative experiment (out of four) is shown. Also shown is an immunoblot of TSG101 and actin levels in COS-1 cells co-transfected with CD4 and siRNA directed against either TSG101 or LacZ. (C) COS-1 cells co-expressing CD4 and either GFP or Vps4E228Q-GFP were treated as described in (A). Data from one representative experiment (out of three) is shown. (D) COS-1 cells were transfected with siRNA directed against either TSG101 or LacZ, serum-starved, and treated with 50 ng/ml EGF for 10 min. The cells were then washed, reincubated with fresh media for 0 or 3 hours, lysed and analysed by SDS-PAGE and Western blotting with an anti-pEGFR antibody. The percent of undegraded pEGFR remaining at 3 hour was determined for the two conditions. Error bars represent SD between duplicates for each condition. (E) COS-1 cells were transfected with GFP or Vps4E228Q-GFP, serum-starved, and allowed to internalize EGF for 10 min. The cells were then washed, reincubated with fresh media for 3 hours and analysed for GFP fluorescence, or endogenous phosphoEGFR by immunofluorescence. The percentage of GFP positive cells with pEGFR remaining at 3 hours was determined for the two conditions. Error bars represent SD between duplicates for each condition.

COS-1 cells expressing exogenous CD4 were metabolically labeled with ^35^S-Met/Cys-translabel for 10 minutes, then chased in non-radioactive medium in the presence or absence of PMA. PMA induced a significant decrease in CD4 levels over a period of 6 hours (Fig. 4A). The experiment was then repeated in cells depleted of endogenous TSG101 using siRNA. At early time points, CD4 degradation was slightly attenuated in TSG101 depleted cells (Fig. 4B, 2 hour time point). However, by 6 hours, CD4 was degraded as efficiently in TSG101-depleted cells as in control cells. Under similar conditions of TSG101 knockdown, EGF induced EGFR degradation was dramatically inhibited (Fig. 4D).

In order to determine whether the AAA-type ATPase Vps4 plays a role in PMA-induced CD4 downregulation, a dominant negative form of Vps4 (Vps4E228Q-GFP) was co-expressed with CD4. At early time points, CD4 degradation was slightly attenuated, but by 6 h, Vps4E228Q-GFP expressing cells had degraded CD4 as efficiently as cells expressing GFP (Fig. 4C). In contrast Vps4E228Q-GFP strongly inhibited EGF-induced EGFR degradation (Fig. 4E). Taken together, these findings suggest that PMA-induced CD4 degradation may be partially dependent on TSG101 and Vps4 function and only during early times after exposure to PMA. With prolonged PMA treatment, CD4 degradation can proceed in the absence of functional TSG101 and Vps4.

### Expression of HIV-1 Gag does not affect PMA-induced CD4 degradation

**Figure 5:**
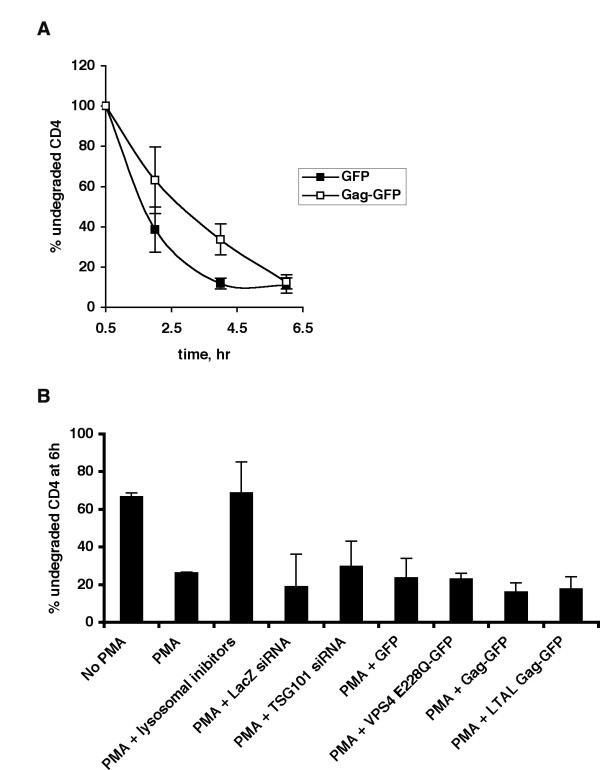
**HIV-1 Gag does not attenuate PMA-induced CD4 downregulation**. (A) COS-1 cells co-expressing CD4 and either GFP or Gag-GFP were treated as described in Fig. 5A. Data from one representative experiment (out of three) is shown. (B) Data shown represents the mean ± SD of % undegraded CD4 remaining after 6 hours of incubation with PMA in COS-1 cells under various conditions as indicated (n ≥ 2).

We next determined whether expression of HIV-1 Gag impinges on the ESCRT-independent downregulation of CD4. Gag expressing cells, like TSG101-depleted and Vps4E228Q overexpressing cells, exhibited an initial slowdown in the rate of CD4 degradation, which was completely overcome upon prolonged exposure to PMA (Fig. 5A). In contrast, lysosomal inhibitors (ammonium chloride and chloroquine) clearly inhibited CD4 degradation (Fig. 5B).

A summary of all the measurements of PMA-induced CD4 degradation represented as the percent of undegraded CD4 remaining after 6 hours of PMA treatment is shown in Fig. 5B. PMA-induced CD4 degradation was not affected by either depletion of endogenous TSG101 or by overexpression of Vps4E228Q or HIV-1 Gag, suggesting that under these conditions, lysosomal degradation of CD4 can proceed in the absence of ESCRT-I and Vps4. Taken together, our studies clearly demonstrate that downregulation of CXCR4, but not CD4, is attenuated by HIV-1 Gag-mediated recruitment of ESCRT complexes. Thus the ability of HIV-1 Gag to impinge on the cellular endocytic pathway is selective, attenuating only ESCRT-dependent processes in this pathway.

## Discussion

In this study, we show that HIV-1 Gag, as well as TSG101, differentially affect the kinetics of downregulation of the HIV-1 co-receptors CXCR4 and CD4. SDF-1-induced CXCR4 downregulation was sharply reduced when TSG101 function is inhibited, while PMA-induced CD4 downregulation was relatively unaffected. Depleting TSG101 using siRNA directed specifically against TSG101 has been shown to result in a reduction in the cellular levels of the other two components of the ESCRT-I complex, Vps28 and hVps37 [11]. Consequently, TSG101-depleted cells have fewer functional ESCRT-I complexes. Thus, our observations imply that CXCR4, but not CD4, is dependent on the ESCRT-I complex for its lysosomal degradation.

Divergent receptors, such as the Epidermal Growth Factor Receptor (EGFR) and the Toll-like receptor, TLR4, have been shown to be dependent on Hrs, ESCRT-I and Vps4 for their transport from early endosomes to late endosomes to lumenal vesicles in the MVB [3,38]. However, this is not the only route into the MVB. For example, sorting and lysosomal degradation of the Delta Opioid Receptor (DOR), a GPCR, is Hrs and Vps4 dependent but does not require TSG101 (ESCRT-I) [21]. Another recent study showed that lysosomal sorting of the GPCR, PAR-1 (Protease-activated receptor-1) does not require Hrs or TSG101 [39]. Moreover, MVB sorting of the melanosome protein Pmel17 is completely independent of any of the members of the 'Vps' family of proteins [40]. It is also important to note that not all ESCRT components are essential for HIV-1 budding and release. For example, depletion of ESCRT-II components or of AIP1/Alix has minimal effects on HIV-1 budding [6,41]. These studies clearly indicate that protein sorting and MVB biogenesis are complex processes involving multiple points of entry and regulation.

Besides modulating the levels of receptors at the cell surface, the cellular endocytic pathway plays a major role in the attenuation of ligand-induced receptor-mediated signaling [42]. For EGFR and other Receptor Tyrosine Kinases, sequestration of the activated receptor into the internal vesicles of the MVB is required to shut down signaling. This process occurs 2–3 hours after ligand binding. Activated EGFR remains in early and late endosomes during this time and is capable of mediating continued intracellular signaling [43]. We previously showed that expression of HIV-1 Gag increases the amount of EGFR that remains in endosomes after EGF addition [14]. As a consequence, Gag expressing cells exhibit hyperactivated and prolonged MAP kinase signaling. Increased MAP kinase activation is advantageous to the virus as it has been shown to enhance HIV-1 replication and infectivity [44,45].

In contrast, GPCR-mediated signaling is attenuated within minutes after ligand binding. CXCR4, like most GPCRs, is desensitized rapidly through the action of GPCR kinases (GRKs), which phosphorylate the receptor on several C terminal Ser/Thr residues [19]. Phosphorylation of CXCR4 promotes binding of β-arrestins, which sterically hinder the association of heterotrimeric G proteins with the receptor and thereby block signal transduction [46]. The increase in intracellular CXCR4 induced by expression of HIV-1 Gag did not result in a change in SDF-1 mediated CXCR4 signaling, as judged by MAP kinase activation (Fig 2C). This finding is consistent with known GPCR biology and confirms that internalized CXCR4 in Gag expressing cells is desensitized and does not signal.

β-arrestin binding to GPCRs also serves to recruit components of the endocytic machinery including clathrin and AP-2, thereby mediating the internalization of the receptor [47]. Following internalization, CXCR4 colocalizes with Hrs-positive endosomes [20]. While Hrs and Vps4 have been implicated in CXCR4 downregulation [20], no role for TSG101 or ESCRTs had been established in this process until now. Our data (Figs 1, 2) strongly suggest that SDF-1 induced CXCR4 downregulation is TSG101 and ESCRT-I dependent. Given that HIV-1 Gag competes with Hrs for TSG101 *in vitro *[48], and that overexpression of TSG101-binding regions of Hrs inhibits HIV-1 release [49], we hypothesized that expression of Gag would compete for TSG101 binding and function *in vivo*. Our observations that TSG101/ESCRT-I dependent downregulation of CXCR4 (Figs 2, 3) and EGFR [14] are attenuated in HIV-1 Gag expressing cells indicate that Gag functionally depletes the ESCRT complexes, thereby interrupting other ESCRT-dependent pathways in the cell.

Two other HIV-1 proteins, Nef and Env, have been shown to interact with or regulate CXCR4. Thus, an important question is whether levels of CXCR4 are altered in the context of an HIV-1 infected cell. Multiple studies have addressed this issue, primarily by quantitating the amount of cell surface CXCR4. A recent study reported that HIV-1 Nef induces downregulation of CXCR4 from the cell surface of infected cells [50]. The authors propose that Nef-mediated CXCR4 downregulation may protect against superinfection. Superinfection is detrimental to viral replication because the accumulation of unintegrated viral DNA results in the induction of cytopathic effects in the host cell [51]. However, multiple other studies have shown that HIV-1 Nef does not downregulate cell surface levels of CXCR4 [52-54] and that maximal protection from superinfection involves an unidentified mechanism that is independent of CXCR4 downregulation [54]. Similarly, we observed no change in cell surface levels of CXCR4 in HIV-1 Gag-expressing cells (Fig. 3F). In contrast, others have, in some cases, seen an upregulation in cell surface expression of CXCR4 in HIV-1 infected CD4+ T cells [55]. SDF-1 induced CXCR4 signaling could potentially be beneficial to viral replication since it results in the activation of transcription factors such as NFκB [16], which are known to increase HIV-1 LTR promoter activity [44]. It is also important to note that HIV-1 Env protein (shed from virus particles or infected cells) can bind to CXCR4 and thereby trigger apoptotic signals. However, CD4 and CXCR4 expression are both required for apoptotic signaling by Env in CD4 T cells [56]. Since CD4 is efficiently removed from the surface of productively infected cells (see below), only uninfected/bystander CD4 T cells express both CD4 and CXCR4 and are therefore susceptible to Env-induced apoptosis [56].

Thus, CXCR4 downregulation might not be essential for HIV-1 replication. We speculate that during the late stages of the viral life cycle when mostly structural proteins such as Gag are expressed, SDF-1 induced CXCR4 downregulation is attenuated resulting in the accumulation of densensitized CXCR4 within intracellular compartments. These receptors could, in the long run, contribute to maintaining or replenishing the cell surface levels of CXCR4 in HIV-1 infected cells.

Unlike SDF-induced CXCR4 downregulation, Gag expression had little to no effect on PMA-induced CD4 downregulation (Fig. 5). PMA is a phorbol ester that binds to and activates protein kinase C (PKC) [57]. PKC is normally activated upon binding of antigen to the T-cell receptor and its associated CD4 [58]. Activated PKC phosphorylates CD4 on its cytoplasmic tail and induces CD4 internalization and lysosomal degradation [25,59]. Several studies have shown that PMA treatment mimics the mechanism of antigen induced CD4 downregulation [23,60]. Surprisingly, little is known about how internalized CD4 gets sorted into the internal vesicles of the MVB prior to lysosomal degradation. In the present study, we show that PMA-induced CD4 downregulation can occur efficiently in the absence of functional ESCRT-I and Vps4 (Fig. 4) and that expression of HIV-1 Gag has no effect on this process (Fig. 5). These findings indicate that Gag affects only ESCRT-dependent processes. We therefore predict that lysosomal degradation of CD4 should not be impeded by Gag in an HIV-1 infected cell. Indeed, loss of cell surface CD4 is a hallmark of HIV-1 infection [51]. After virus entry, it is essential that HIV-1 efficiently downregulates CD4 for multiple reasons. CD4 downregulation is important to prevent superinfection of the infected cell [61]. In addition, cross-linking of CD4 in the absence of T cell receptor activation results in the generation of non-proliferative or apoptotic signals [62]. Viral transcription is also inhibited under these conditions [63]. Many studies have also reported that cells overexpressing CD4 exhibit a drastic inhibition of virion release [64,65]. Moreover, the presence of CD4 at the cell surface appears to significantly reduce the infectivity of released virions [66,67]. Exactly how CD4 exerts these effects is unclear, but these observations establish the critical need for HIV-1 to downregulate CD4 in infected cells. Three different viral proteins, Nef, Env and Vpu have evolved to ensure that cell surface CD4 is downregulated soon after entry (by the early protein Nef) and that transport of newly synthesized CD4 to the cell surface at late stages of infection is blocked (by late proteins Env and Vpu) [51,68]. Thus, by the time Gag proteins are expressed in an infected cell, most of the surface CD4 has already been downregulated by Nef.

## Conclusion

Our observations indicate that expression of HIV-1 Gag functionally depletes cellular ESCRT complexes. As a consequence, Gag expression modulates ESCRT-dependent but not ESCRT-independent receptor sorting pathways in the host cell. These findings are likely to be highly relevant to HIV-1 pathogenesis as they shed light on the mechanisms used by HIV-1 proteins to dysregulate normal cell physiology and to potentiate viral replication.

## Methods

### Antibodies and reagents

The following antibodies were purchased as indicated: anti-HA monoclonal (12CA5) and polyclonal antibodies and anti-CXCR4 polyclonal antibody, Sigma (St. Louis, MO); anti-phosphoERK monoclonal antibody and anti-actin, anti-GFP, anti-phosphoEGFR and anti-ERK polyclonal antibodies, Santa Cruz Biotechnology (Santa Cruz, CA); anti-TSG101 monoclonal antibody, GeneTex. Inc (San Antonio, TX); anti-EEA1 monoclonal antibody, BD Transduction Laboratories (San Diego, CA); anti-CD63 monoclonal antibody, Cymbus Biotechnology Ltd (UK); Biotinylated anti-mouse CXCR4, BD Pharmingen (San Jose, CA); anti-β-Gal, Promega, (Madison, WI). Monoclonal anti-CD4 (leu3A) was used to immunoprecipitate CD4. A homemade rabbit anti-p24CA antiserum was used to detect Gag. Trans ^35^S-label was purchased from MP Biomedicals (Irvine, CA). SDF-1α was obtained from Peprotech Inc. (Rocky Hill, NJ). PMA, Cycloheximide and Chloroquine were purchased from Sigma (St. Louis, MO). Ionomycin was obtained from Calbiochem-EMD Biosciences (La Jolla, CA). EGF was obtained from Oncogene Research Products (San Diego, CA). Streptavidin-PE was a kind gift from Dr. Lionel Ivashkiv (Hospital for Special Surgery, and Weill Graduate School of Medical Sciences of Cornell University, New York, NY).

### DNA plasmids and siRNA

The Rev independent Gag expression vectors pGag-GFP and LTAL Gag-GFP have been described elsewhere [14,69]. pEGFP-TSG101 (full length) was a kind gift from Dr. Stanley Cohen (Stanford University, Stanford, CA). 3xHA-CXCR4 was obtained from the UMR cDNA resource center (University of Missouri-Rolla, Rolla, MO). pEGFP-N2 CD4 (full length) was a kind gift from Dr. John Wills (Pennsylvania State University College of Medicine, Hershey, PA). VPS4E228Q-GFP was a kind gift from Dr. Uta von Schwedler (University of Utah School of Medicine, Salt Lake City, UT). siRNAs directed against human TSG101 (5'-CCUCCAGUCUUCUCUCGUCTT-3') and LacZ were obtained from the High Throughput Screening Core Facility at Memorial Sloan Kettering Cancer Center (New York, NY).

### Cell culture and transfections

COS-1 cells were maintained in DMEM/10% fetal bovine serum (FBS). For transfections, COS-1 cells were seeded to approximately 50% density and transfected the next day with 2 to 6 μg plasmid DNA using Lipofectamine 2000 (Gibco BRL Life Technologies, Carlsbad, CA). For TSG101 knockdown experiments, COS-1 cells were seeded to approximately 30% density and transfected the next day with 25 to 50 nM TSG101 or LacZ siRNA and 0.5 to 1 μg plasmid DNA using Lipofectamine 2000 according to the manufacturer's recommendations. 24 hours later, the cells were transfected again with 25 to 50 nM TSG101 or LacZ siRNA. Cells were harvested and analyzed for effects of TSG101 knockdown the next day. Jurkat T cells were maintained in RPMI/10% fetal bovine serum (FBS) supplemented with 2 mM Glutamax (Gibco). For expression of exogenous proteins, 1 × 10^5 ^Jurkat cells were transduced with lentiviral vectors encoding either LacZ, Gag-GFP or LTAL Gag-GFP at a multiplicity of infection (MOI) of 10.

### Lentivirus production and titration

TheViraPower™ Lentiviral Directional TOPO^® ^Expression Kit was purchased from Invitrogen (Carlsbad, CA). Rev-independent wild type HIV-1 Gag GFP and the late domain mutant, LTAL Gag GFP were TOPO^®^-cloned into pLenti6/V5-D-TOPO^®^-plasmid as per the manufacturer's instructions. After sequence verification, the pLenti6/V5-D-TOPO^®^-expression plasmid was cotransfected with the ViraPower™ Packaging Mix into the 293FT cell line to produce lentivirus. 48 hours later, the viral supernatant was harvested and titered as follows. Three different dilutions (1/100, 1/1000 and 1/10000) of the viral supernatant were used to transduce 1 × 10^5 ^Jurkat T cells, in the presence of 6 μg/ml Polybrene^®^. On the next day, the transduced Jurkat cells were pelleted at 150 × g and resuspended in fresh RPMI/10% FBS/Glutamax. 48 hours following transduction, Jurkat cells were analyzed by flow cytometry (BD Biosciences FACSCalibur flow cytometer); over 100,000 cells were analyzed for GFP expression. The viral titers were calculated as follows Transduction units/ml = (average cell number at time of transduction × % of GFP-positive cells)/100 × dilution factor [70]. The optimal multiplicity of infection (MOI) to be used was determined by transducing Jurkat T cells at various MOIs (0, 1 and 10), changing the media the next day, then analyzing the cells by flow cytometry for GFP expression after another 24 hours.

### Immunofluorescence microscopy

Transfected COS-1 cells grown on coverslips were serum starved for 16 hours, and then processed 48 hours post-transfection [69]. HA-CXCR4 downregulation experiments were performed as previously described [20]. Briefly, cell surface receptors were labeled with an anti-HA antibody for 1 hour on ice, washed twice with cold PBS, then incubated in DMEM/10% FBS with or without 100 nM SDF-1α for 3 hours at 37°C. After fixation and permeabilization, cells were incubated with an Alexa Fluor^® ^594-conjugated secondary antibody (Molecular Probes, Eugene, OR). Cells were then washed four times for 5 minutes each with PBS, and mounted on microscope slides. For nuclear staining, HOECHST dye was added to cells during the first PBS wash after secondary antibody incubation. EGFP fluorescence was visualized directly. Laser scanning confocal microscopy was performed on a Zeiss LSM510 confocal microscope equipped with an Axiovert 100 M inverted microscope using a 63×, 1.2-numerical-aperture (NA) water immersion lens for imaging as previously described [69]. We first determined the percentage of cells that were initially expressing CXCR4 (X4_t = 0h_). Then we determined the percentage of cells that had CXCR4 signal remaining after 3 hours of incubation with SDF-1 (X4_t = 3h_). The degradation efficiency was calculated as: [1 - (X4_t = 3h_/X4_t = 0h_)] × 100.

For colocalization experiments, cells were incubated with monoclonal anti-EEA1 or monoclonal anti-CD63 antibody following the 3 hour SDF treatment. Cells were then stained with the Alexa Fluor^® ^594-conjugated goat anti-rabbit antibody (for HA-CXCR4) and a Cy5-conjugated anti-mouse antibody (for EEA1 or CD63). Colocalization was measured on a pixel-by-pixel basis using MetaMorph software (Universal Imaging Corp., Downingtown, PA). EGF-induced EGFR downregulation experiments were performed as previously described [14].

### CXCR4 signaling

Transfected COS-1 cells grown on 60 mm dishes were serum starved for 16 hours. Cells were then treated with 100 ng/ml SDF for 0, 2, 10, 30 or 60 minutes at 37°C. At each time point, cells were lysed in RIPA buffer (150 mM NaCl, 1% NP-40, 0.5% Deoxycholic acid, 0.1% SDS, 50 mM Tris pH 8.0) containing leupeptin (10 ug/ml), aprotinin (10 ug/ml), AEBSF (0.25 mg/ml), NaF (1 mM) and Na_3_VO_4 _(0.5 mM). Lysates were clarified at 20,800 × g in an Eppendorf centrifuge for 10 min at 4°C. Western blotting was performed using the indicated antibodies. Proteins were detected using horseradish-peroxidase conjugated secondary antibodies and ECL Western blotting detection reagents using the manufacturer's instructions.

### CXCR4 downregulation in Jurkat T cells

1 × 10^5 ^Jurkat T cells were pelleted at 150 × g, and incubated in 50 μl of RPMI/10% FBS/2 mM Glutamax containing 50 μg/ml cycloheximide for 15 minutes at 37°C. 50 μl of the same medium, either with or without 100 nM SDF, 50 ng/ml PMA and 800 ng/ml Ionomycin was then added and the cells were incubated at 37°C for 0, 1.5, 3, 6 or 9 hours. At each time point, cells were harvested, washed once in PBS, lysed in 2 × SDS sample buffer by sonication and proteins were resolved by SDS-PAGE. Endogenous CXCR4 was detected using an anti-CXCR4 rabbit polyclonal antibody while expression of the Gag and β-Gal proteins was determined using anti-p24CA and anti-β-Gal antibodies respectively. Equal loading of proteins was confirmed by detecting actin using an anti-actin goat polyclonal antibody. Western blots were analyzed by chemiluminescence (Pierce) and exposed to Biomax MR films (Kodak, New Haven, Conn). Films were scanned using an HP scanner and quantified using ImageGauge Version 4.1 (FujiFilm).

### Detection of Cell Surface levels of CXCR4 in Jurkat T cells

48 hours post transduction, Jurkat T cells were pelleted at 150 × g, and incubated with a biotinylated anti-CXCR4 antibody or an isotype-matched control antibody (1 μg per 10^6 ^cells) for 30 minutes, on ice. Cells were then washed in staining buffer (PBS/1% FBS), incubated with Streptavidin-PE (1:500) for 30 minutes on ice, washed and resuspended in staining buffer. PE fluorescence was analyzed by flow cytometry (BD Biosciences FACSCalibur flow cytometer).

### Metabolic labeling and CD4 downregulation

Transfected COS-1 cells were metabolically labeled as described previously [71], using 50 μCi/ml Trans ^35^S-label. The cells were pulse-labeled for 10 minutes at 37°C, then chased in DMEM/10%FBS containing 100 μM cysteine and methionine, with or without 50 ng/ml PMA, for 0.5, 2, 4 and 6 hours. At each time point, cells were washed once with STE then lysed in RIPA buffer (150 mM NaCl, 1 mM EDTA, 0.1% SDS, 0.5% deoxycholate, 1% Triton-X 100, 10 mM Tris, pH 7.4) containing protease inhibitors (10 μg/ml aprotinin, 10 μg/ml leupeptin and 0.25 mg/ml AEBSF). The lysates were clarified at 100,000 × g in a Beckman TL-100 ultracentrifuge for 15 minutes at 4°C. About 20 μl of the clarified lysate was kept aside (sample before IP) and the rest of the lysate was then incubated overnight at 4°C with 2.5 μg mouse anti-CD4 (Leu3a) antibody and 15 μl protein A/G plus agarose beads (Santa Cruz, CA). The beads were washed three times in RIPA buffer containing protease inhibitors. Immunoprecipitated CD4 was eluted from the beads by adding 2 × SDS sample buffer. The beads were boiled and pelleted, and the eluted sample was analyzed by SDS-PAGE and phosphorimaging using the Fluoro image analyzer FLA-5000 (FujiFilm, Stamford, CT). Images were quantified using ImageGauge version 4.1 (FujiFilm). Samples before IP were also subjected to SDS-PAGE and Western blotting to determine levels of actin and other exogenous proteins in the samples. To test the effect of lysosomal inhibitors on PMA-induced CD4 downregulation, the experiment described above was performed using medium containing 50 mM NH_4_Cl and 100 μM chloroquine.

## Abbreviations

ESCRT (endosomal sorting complex required for transport); multivesicular body (MVB); TSG101 (Tumor Susceptibility Gene 101); EGF Receptor (EGFR); SDF-1 (stromal cell-derived factor).

## Competing interests

The author(s) declare that they have no competing interests.

## Authors' contributions

RRV performed all experiments and wrote the majority of the manuscript. RRV and MDR jointly conceived the rationale for the study and designed the experiments. MDR wrote part of the manuscript, and edited the entire manuscript. Both authors read and approved the final manuscript.
